# Multiple neural tube defects: rare developmental anomaly with an elusive embryological explanation

**DOI:** 10.1259/bjrcr.20230005

**Published:** 2023-07-10

**Authors:** Pallavi Sinha, Atin Kumar, Manisha Jana, Devasenathipathy Kandasamy

**Affiliations:** 1 Department of Radiodiagnosis and Interventional Radiology, All India Institute of Medical Sciences, New Delhi, India

## Abstract

Neural tube defect is a congenital anomaly resulting from the failure of fusion of the neural folds in the midline which occurs in the third and the fourth week of embryonic development. These defects can occur at any of the three embryological stages—gastrulation, primary neurulation and secondary neurulation. Presence of neural tube defects at multiple (two or more) sites along the craniospinal axis is an extremely rare anomaly and the management depends on clinical as well as imaging findings. These multiple defects are not well explained by the “Zipper closure” theory and can be better explained by the “Multisite closure theory”, which will be highlighted in this manuscript. Few of these multiple site anomalies cannot be fully explained even by the multisite closure theory and more research is needed to decipher this entity.

## Introduction

Neural tube defect (NTD) is a congenital developmental anomaly that occurs due to the failure of fusion of the neural folds in the midline. This primary failure of the development of normal neural tube results in secondary abnormal development of the mesoderm responsible for the formation of the bones and muscles that cover the underlying neural structures, resulting in various types of neural tube defects. Its clinical presentation is quite variable and imaging plays an important role in deciphering the type of NTD which is critical for further management. The presence of neural tube defects at multiple (two or more) sites along the craniospinal axis is an extremely rare anomaly, comprising less than 1% of the total reported cases of NTD.^1^ We came across two cases that defy the age-old ‘Zipper closure’ theory. We reviewed the literature on the embryogenesis of multiple NTD, which can be better illustrated by the more recent multisite closure theory. Our first case had both cervical myelomeningocele (MMC) and thoracic lipomyelocele; whereas the second case had segmental diastematomyelia at cervical and lumbar region with myelocele of the split cord at sacral level, and a normal intervening thoracic spinal cord.

## Case report

### Case 1

**Figure 1.**
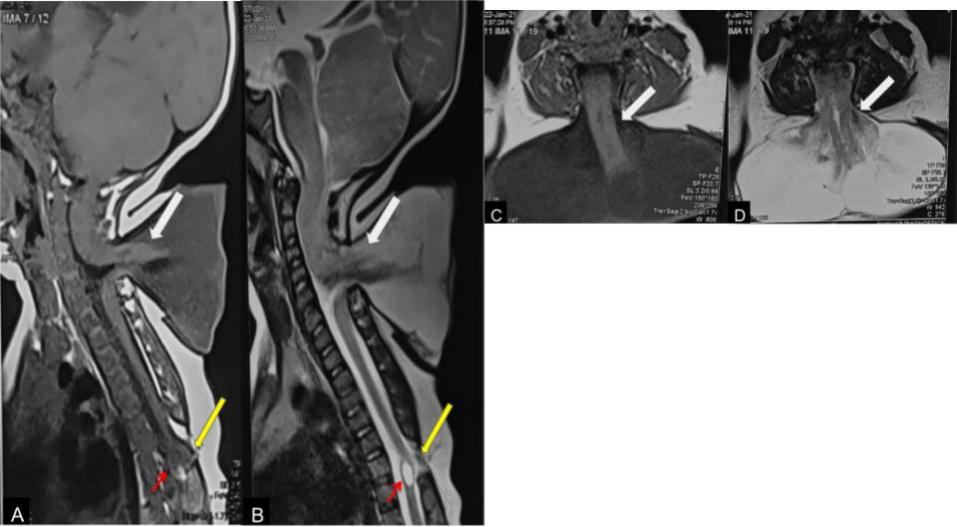
Sagittal *T*_1_W (**A**) and *T*_2_W (**B**) images show the cervical meningomyelocele with a defect in posterior elements of the cervical vertebrae (**C4–C6**) and herniation of neural elements in the CSF filled sac (white arrow). Axial *T*_1_W (**C**) and *T*_2_W (**D**) images at the same level also show the herniation of neural elements (white arrow) outside the spinal canal. In addition, there is another site of defect in the posterior elements of T6–T7 vertebrae (yellow arrow in A, B) with posteriorly displaced spinal cord, thin hypointense band reaching up to the subcutaneous fat and an intraspinal cystic lesion (red arrow) suggestive of lipomyelocele. The neural placode was seen outside the spinal canal, suggestive of cervical myelomeningocele. Another defect was noted in the posterior element in the thoracic spine at the level of T6–T7 vertebrae. The spinal cord at this level was displaced posteriorly, which was in contact with the subcutaneous fat suggestive of lipomyelocele. A focal syrinx was noted at the same level. CSF, cerebrospinal fluid.

An 8-month-old girl presented with a swelling in the posterior aspect of the neck and upper back since birth, with a history of occasional dribbling of urine. On examination, there was a 9 × 5 × 6 cm swelling in the cervical and upper back region. The swelling was cystic in consistency, transilluminant and no obvious leak of fluid was noted. The anal tone was normal and the bladder was not expressible. Power in bilateral lower limbs was 3/5. There were no clinical signs of hydrocephalus. On examination of the spine, in addition to the swelling in the neck, a defect was also palpable in the thoracic spine. On MRI, there was a well-defined cerebrospinal fluid-filled structure in the cervical region protruding from the defects in the posterior elements of C4 to C7 vertebrae (Figure 1).

### Case 2

**Figure 2.**
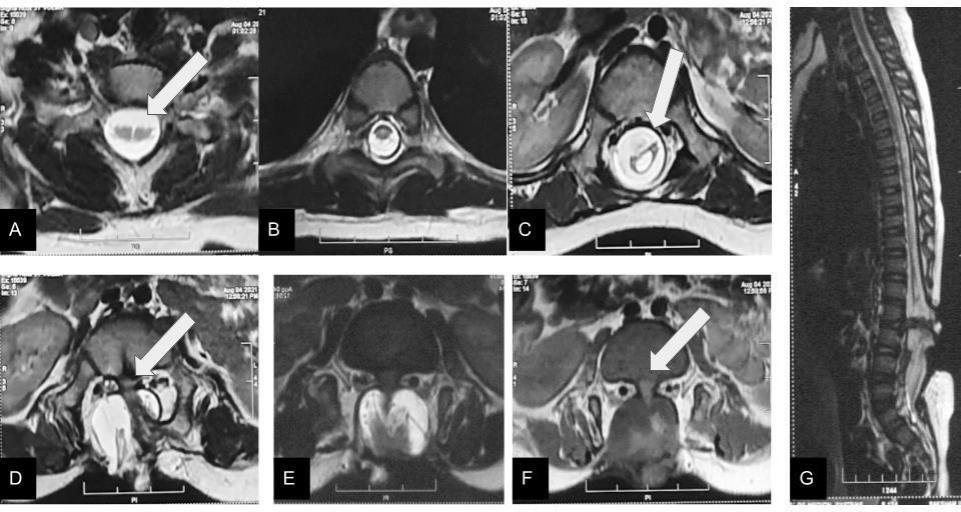
Axial *T*_2_W (**A**) image shows Pang Type 2 split cord malformation (white arrow) at cervical level. Axial *T*_2_W (**B**) image shows single cord with normal signal intensity at the thoracic level. Images (**C–F**) show single cord with syrinx in lumbar region (white arrow in C) and a Pang Type 1 split cord malformation at lumbosacral region with an intervening bony septum (white arrow in D). The cord is tethered, low lying and shows herniation of neural elements through defect in the posterior elements of lumbar vertebrae suggestive of myelocele. T2 sagittal image (**G**) shows the NTD is flat. NTD, neural tube defect.

An 8-month-old girl presented to AIIMS, New Delhi with flaccid paraplegia since birth, absent deep tendon reflexes of lower limbs and an upper motor neuron type bladder. On examination, there was a scar over the lower back with a palpable defect in the sacral spine. On imaging (Figure 2), there was split cord malformation in the cervical level with both the hemicords in a single sac suggestive of Pang Type 2 split cord malformation. The thoracic cord was normal on imaging. There was Pang Type 1 split cord malformation in the lumbar spinal cord with an intervening bony spur with both the hemicords in separate dural sacs. There was a defect in the posterior elements of sacral vertebrae with herniation of both the cords and the neural placode through the defect. The anterior subarachnoid space was expanded but the posterior was not and the lesion was flat suggestive of myelocele. Informed consent was obtained from the parents of both cases.

## Discussion

Dysraphism means the persistence of continuity between the posterior neural ectoderm and cutaneous ectoderm. Cranial dysraphism results from failure of cranial neural tube closure and includes anencephaly and encephaloceles. Spinal cord development encompasses three embryological stages–gastrulation, primary neurulation and secondary neurulation.

Congenital abnormalities of the spine and spinal cord occur due to aberration from normal pathways at any stage of development. NTD at multiple levels is a very rare anomaly and the Zipper model which can explain the common types of NTD cannot explain the embryology of NTD at multiple levels. A recently proposed Multisite closure theory better untangles the embryological basis of these rare defects.

## Theories for neural tube closure

### Zipper model

The conventional theory on the etiogenesis of MMC is based on the Zipper model which states that neural tube closure is a continuous, bidirectional process.^1^ NTD results from defective closure of the cranial (around 24 day) and caudal neural pores between 26 and 28 days of gestation. However, if the neural tube closure begins in the mid-cervical region and progresses in a zipper-like fashion in both cranial and caudal directions, it cannot explain the presence of neural tube defect at the sites other than anterior and posterior neuropores.

### Multisite closure model

**Figure 3.**
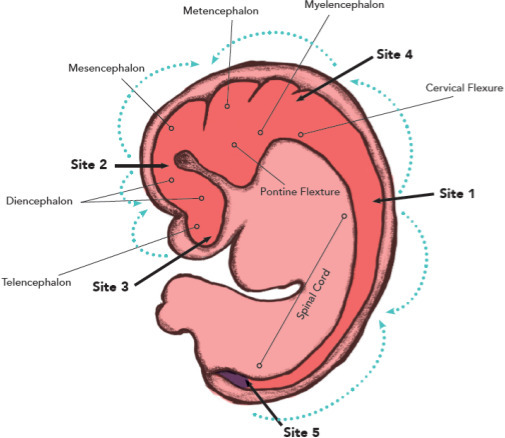
Schematic diagram illustrating the various sites of closure according to the “multisite” closure model. The first site of closure (closure 1) proceeds bidirectionally from the mid-cervical region to the level of L2. Closure 2 is initiated at the boundary of prosencephalon and mesencephalon, advances bidirectionally to meet site three cranially and terminates caudally at the cranial aspect of the rhombencephalon. Closure 3 proceeds cranially from the stomatodeum and reaches its destination which is the cranial end of closure site 2. Closure 4 starts at the caudal end of the rhombencephalon and meets the caudal aspect of site two by advancing in a rostral direction. Closure 5 begins at the caudal end of the neural tube, advances in rostral direction and leads to the closure of the neural tube (S2 to L2) and proceeds rostrally. Closure below S2 occurs by the process of secondary neurulation. Both the anomalies in our case one are attributed to aberration in the initiation of closure, that is the site of closure 1.

According to this theory, the neural tube closure in the embryo commences at five sites. NTDs occur at the “collision sites'' of neural tube closure with opposing closure directions^2–4^ (Figure 3).

The second case had split cord malformation, which plausibly might have resulted due to the adhesions between ectoderm and endoderm, leading to the genesis of an accessory neurenteric canal. An endomesenchymal tract encompasses the neurenteric canal that bisects the developing notochord, resulting in the formation of two hemineural plates.^5^ The final configuration and orientation of hemicord depend upon the altered state of the split neural tube, the constituent components of the endomesenchymal tract and the median septum.

The majority of neural tube defects can be attributed to the failure of fusion of one of the closures or their contiguous neuropores. It has been earlier proposed that anencephaly and spina bifida result from defective primary closure and encephaloceles occur due to post-closure defects. According to multisite closure theory, the laterally situated encephaloceles are postclosure defects, however, the midline encephaloceles are better illustrated by a primary closure defect. This multisite theory unravels the puzzle of multiple NTDs better than the Zipper model. There can be complete or partial failure of closure. Pathologies manifesting due to complete failure of closure are meroacranium, holoacranium, facioacranium and occipital cranioschisis. Pathologies attributed to partial failure of closure are various types of encephalocele, MMC at various levels and lipomas.^6^

One of the shortcomings of the multisite theory is the rarity of double or multiple neural tube defects. However, it is supported by the fact that multiple NTDs are usually incompatible with life, hence rarely encountered.^5^ The other shortcoming of multisite theory has been the etiopathogenesis of cervical MMC which has several unique features such as intact neurological functions; full-thickness skin cover; less common association with Chiari malformation type II; the herniated content is a neuroglial stalk that arises from the dorsal surface of the cervical cord and causes the tethering and not the neural placode per se.^5^ These features have been better explained by the limited dorsal myeloschisis theory of Pang and Dias.^7^ According to this theory, there is an uneventful progression of neurulation, except for a thin slip in the dorsal midline. There is a failure of separation of the cutaneous ectoderm and neuroectoderm. Though myofascial tissues develop from a dorsal median stalk of central nervous system tissue, this unseparated tissue remains as the original attachment between the nearly closed neural tube and the still slightly gaping cutaneous ectoderm. The meninges develop circumferentially except for an extension around this stalk. There is formation of MMC by seepage of cerebrospinal fluid into the dural fistula which eventually distends the meningeal sac.

Our first case had cervical MMC associated with myelocele which can not be fully explained with this multisite theory alone but can be explained with the incorporation of limited dorsal myeloschisis theory of Pang and Dias. The second case had diastometamyelia at two isolated levels and lipomyelocele, both of which occur due to completely unrelated mechanisms with insults at different stages of embryogenesis but are commonly associated anomalies. In cases of multiple NTDs, the closure of the ruptured, larger, or most cranial NTD should be undertaken first. Closure of multiple NTDs in the same surgery increases complications so a staged procedure is preferred.^8^

## Learning points:

1. Multiple neural tube defects are not as rare as previously thought.
2. The zipper model of neural tube closure fails to explain both the occurrence of multiple neural tube defects as well as the occurrence of neural tube defects at sites other than that at anterior and posterior neuropores.
3. The multisite closure theory better explains the occurrence of multiple neural tube defects in a child as well as helps to characterise them into those due to complete failure of closure and those due to partial failure of closure.
4. More research is needed as even multisite theory is not able to provide a plausible explanation of cervical meningomyelocele.

## Conclusion

Multiple NTD is an extremely rare entity with an elusive embryological basis. Very few such cases have been reported and despite years of extensive research, the exact pathogenesis of multiple NTD remains unclear. More research in this field is needed to unravel this mysterious anomaly. Early repair, whether single- or multistep procedures are needed to prevent long-term complications.
